# A Treatise on Mediate Auscultation and on Diseases of the Lungs and Heart

**Published:** 1846-07

**Authors:** 


### A Treatise on Mediate Auscultation and on Diseases of the Lungs and Heart. By *R. T. H. Laennec.* Translated by a Member of the College of Physicians. Edited by *Theophilus Herbert,* M.D. With Practical Notes, condensed from the Lectures of *F. H. Ramadge,* M.D. Octavo, pp. 862. London, 1846.

We felt somewhat surprised at perceiving the announcement of a new translation of Laennec's immortal work, inasmuch as the profession is already in possession of one by an accomplished physician, well qualified to do justice to the task. Upon examining the book, however, we have been still more so, for we could not have believed that any man would have had the hardihood to dedicate a translation of the work of this great and scientific physician to a person of the calibre of Dr. Ramadge—yet so it is; and not only this, but preface and notes are filled with the most fulsome adulation of this personage; their writer evidently implying, that if we are to do honour to him who placed in our hands the means of distinguishing diseases of the chest, and left us the best description of their characters, we must surely be prepared to prostrate ourselves before the hero who tells us he can cure the most deadly of them. This is not the occasion we should choose for the exposure of the fallacy of Dr. Ramadge's views, and the curious statements they are attempted to be supported by: but we could not allow his former pupil and friend to bring his name into juxta-position with that of the great Laennec, without protesting against it, as, to say the very least, a piece of gross absurdity.

The translation (by another hand) is executed fairly enough, and several of the notes by Andral are valuable; but why the work was ever published, save for the laudation of the aforesaid Dr. Ramadge, we cannot imagine. To those who may wish to become acquainted with his views, it may prove acceptable, for they are reiterated at every opportunity, fitting or not: but we believe they are too generally acknowledged as erroneous to secure many purchasers on their account.

